# Opportunistic assessment of bone mineral density using computed tomography in pediatric liver transplant recipients

**DOI:** 10.1007/s00431-026-06794-w

**Published:** 2026-02-21

**Authors:** Nurullah Dag, Sevgi Tasolar, Hilal Er Ulubaba, Mehmet Candur, Sezai Yilmaz

**Affiliations:** 1 Department of Radiology, Faculty of Medicine, Inonu University, Elazig Road 15. Km 44280, Battalgazi, Malatya, Türkiye; 2 Department of General Surgery and Liver Transplant Institute, Faculty of Medicine, Inonu University, Elazig Road 15. Km 44280, Battalgazi, Malatya, Türkiye

**Keywords:** Bone health, Pediatric liver transplantation, Computed tomography, Dual-energy X-ray absorptiometry, Hounsfield unit

## Abstract

Pediatric liver transplant (LT) recipients are at increased risk of impaired bone mineral accrual due to chronic liver disease, growth disturbances, and post-transplant metabolic factors. Although dual-energy X-ray absorptiometry (DXA) remains the reference method for evaluating bone mineral density (BMD), opportunistic assessment using routine abdominal computed tomography (CT) has emerged as a potential adjunct tool. This study aimed to evaluate the potential of using routine abdominal CT scans to assess bone health in pediatric LT recipients by comparing lumbar vertebral Hounsfield unit (HU) values with those obtained using DXA. This retrospective study included 62 pediatric LT recipients who underwent both abdominal CT and lumbar spine DXA within a 3-month period. HU values were measured at vertebral levels L1–L4 in both the sagittal and axial planes. DXA *z*-scores were classified according to International Society for Clinical Densitometry pediatric guidelines. Correlation and receiver operating characteristic (ROC) analyses were performed to determine the diagnostic performance of HU values. Patients with low bone mineral density had significantly lower sagittal HU values (*p* < 0.05). Moderate correlation was found between sagittal mean HU and DXA *z*-scores (*r* = 0.429, *p* = 0.003), whereas weaker correlation was observed for axial HU values (*r* = 0.266, *p* = 0.037). ROC analysis demonstrated a moderate discriminative ability for sagittal HU (AUC = 0.713, *p* = 0.016), with an optimal cut-off value of 188 HU yielding 83% sensitivity and 68% specificity.

*Conclusion*: CT-derived HU values from routine abdominal imaging may provide valuable supplementary information on bone health in pediatric LT recipients. However, CT-based bone assessment should be considered a complementary approach, enhancing clinical decision-making by providing supportive quantitative information in scenarios where DXA is unavailable or impractical.

**Table:** 

**What is Known:** • *Pediatric liver transplant recipients are at increased risk of impaired bone mineral accrual, and DXA remains the reference standard for pediatric bone mineral density assessment.* • *In adults, vertebral HU values from routine CT correlate with bone mineral density, but evidence in pediatric populations remains limited.*
**What is New:** • *CT-derived sagittal vertebral HU values showed a moderate correlation with DXA lumbar z-scores in pediatric liver transplant recipients, whereas axial measurements were weaker.* • *A sagittal mean threshold of ~188 HU demonstrated a moderate diagnostic performance for identifying low bone mineral density, supporting opportunistic contrast-enhanced CT as a complementary—not replacement—tool to DXA.*

**What is Known:**

• *Pediatric liver transplant recipients are at increased risk of impaired bone mineral accrual, and DXA remains the reference standard for pediatric bone mineral density assessment.*

• *In adults, vertebral HU values from routine CT correlate with bone mineral density, but evidence in pediatric populations remains limited.*

**What is New:**

• *CT-derived sagittal vertebral HU values showed a moderate correlation with DXA lumbar z-scores in pediatric liver transplant recipients, whereas axial measurements were weaker.*

• *A sagittal mean threshold of ~188 HU demonstrated a moderate diagnostic performance for identifying low bone mineral density, supporting opportunistic contrast-enhanced CT as a complementary—not replacement—tool to DXA.*

## Introduction

Liver transplantation (LT) is a life-saving treatment for acute liver failure and end-stage liver disease in children [1]. Advances in surgical techniques, immunosuppressive therapies, and perioperative care have increased long-term survival rates [2]. However, with increasing life expectancy, long-term morbidities such as metabolic and skeletal complications following transplantation are becoming more significant [3]. As bone metabolism is particularly susceptible to deterioration in growing children, regular follow-up and early assessment of skeletal health are of paramount clinical importance [4].

Metabolic bone disease is a multifactorial complication characterized by a deterioration in bone mineral density (BMD) in patients with chronic liver disease [5]. In pediatric patients, this condition is further complicated by growth failure, malnutrition, vitamin D deficiency, and the effects of immunosuppressive drugs used after LT [6]. Early diagnosis is crucial for preventing fractures, growth disturbances, and permanent skeletal deformities [7]. However, as clinical signs are often subtle, there is a need for reliable, accessible, imaging-based evaluation methods.

Dual-energy X-ray absorptiometry (DXA) is widely regarded as the reference standard method for the assessment of BMD in adult and pediatric populations [8]. In addition to the quantitative determination of bone mineral content, this technique provides an objective assessment in children using age- and sex-adjusted *z*-scores [9]. According to the pediatric guidelines established by the International Society for Clinical Densitometry (ISCD), a *z*-score of − 2.0 or less is categorized as “low bone mineral density for age” [8]. However, DXA is not without its disadvantages, including cost, device availability, sensitivity to body composition, positioning errors, and limited anatomical coverage [10].

Abdominal computed tomography (CT) is frequently used in liver transplant recipients to evaluate the graft and potential complications [11]. During these routine examinations, Hounsfield unit (HU) values measured from the lumbar vertebrae can provide information about BMD. Studies in adult populations have demonstrated a strong correlation between HU values obtained from CT scans and BMD measurements determined by DXA [12]. However, data on this relationship in the pediatric population are limited. Therefore, this study aimed to evaluate the potential of CT in assessing bone health in pediatric liver transplant patients by comparing HU values measured at the lumbar spine with DXA. Our goal was to explore the feasibility of using CT images, which are frequently obtained during routine clinical follow-up, as an opportunistic tool for bone health assessment.

## Materials and methods

### Study design and population

This single-center retrospective study was approved by Inonu University Clinical Research Ethics Committee (2025/8754); given its study nature, informed consent was waived. All procedures were conducted in accordance with the ethical principles of the 1964 Declaration of Helsinki and its later amendments.

Pediatric patients (< 18 years) who underwent LT between January 2012 and July 2024 were retrospectively identified. Among these, patients who had DXA available and underwent contrast-enhanced abdominal CT within 3 months before or after the DXA examination were included. Patient inclusion was primarily determined by the adequacy of CT image quality and the feasibility of reliable vertebral attenuation measurements. Following this CT-based eligibility assessment, corresponding DXA records were subsequently reviewed, and only cases with evaluable lumbar spine DXA data were included in the final analysis. Patients with structural abnormalities or focal lesions of the lumbar vertebrae—such as congenital vertebral malformations, bone cysts, postinfectious or posttraumatic changes, or marrow-infiltrative diseases—were excluded. Children with vertebral compression deformities, previous spinal surgery, metallic implants, or motion artifacts interfering with measurement accuracy were also excluded.

### CT acquisition protocol and HU measurements

All contrast-enhanced CT images were obtained in the venous phase of the abdomen as part of routine post-transplant follow-up examinations. Images were acquired using multi-detector CT scanners, including the Somatom Definition 256-slice system (Siemens Healthineers, Erlangen, Germany) and the Aquilion 64-slice system (Canon Medical Systems, Otawara, Japan; formerly Toshiba Medical Systems), with standardized acquisition parameters: 80–120 kVp tube voltage (adjusted for age and weight), automated tube current modulation, 1 mm slice thickness, and 512 × 512 reconstruction matrix. All images were reconstructed using a standard soft-tissue kernel in both axial and sagittal planes to ensure optimal visualization of vertebral trabecular bone.

**Fig. 1:**
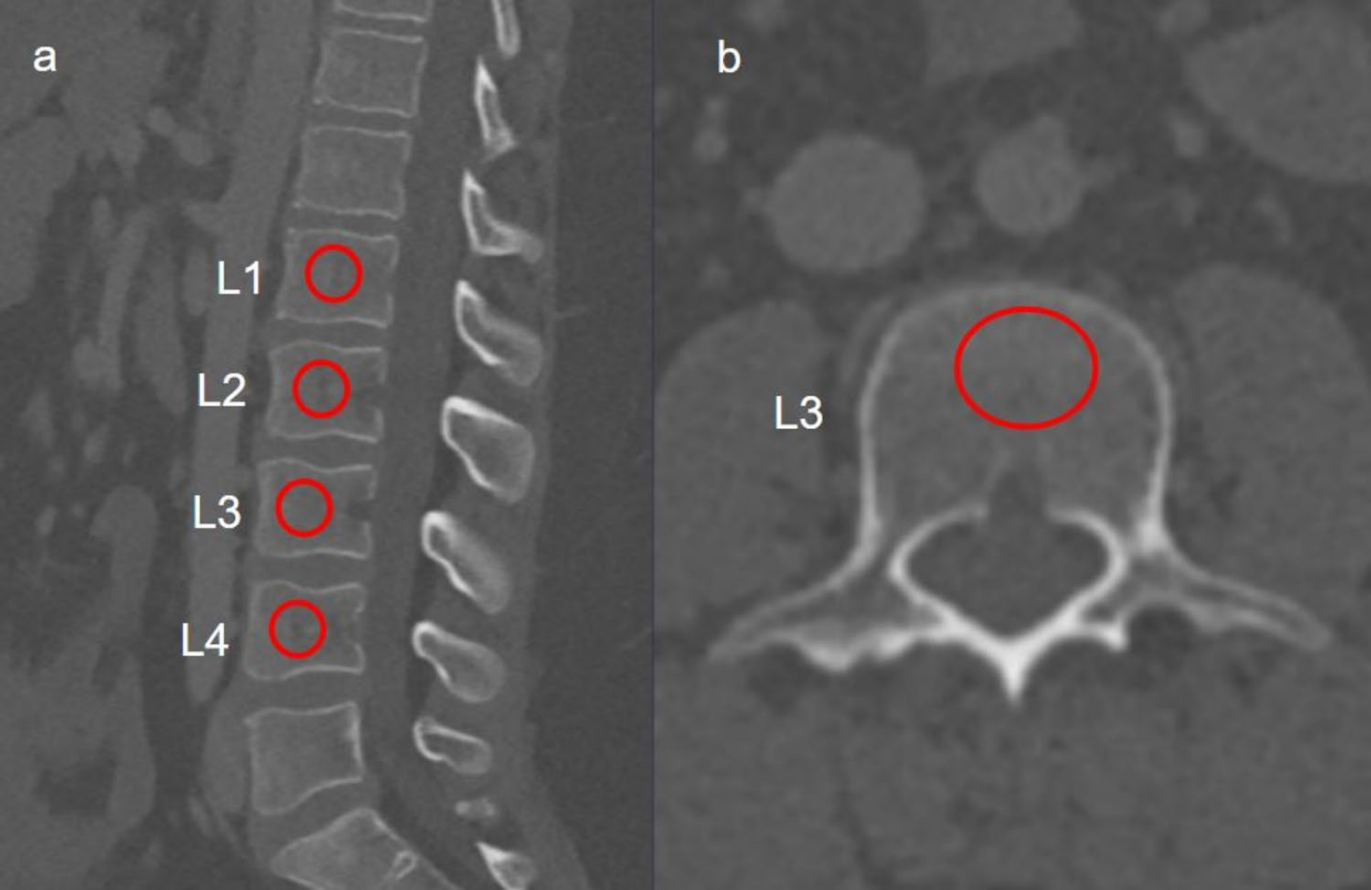
Sagittal (**a**) and axial (**b**) CT images of the lumbar vertebrae demonstrating the measurement of HU values using a circular region of interest (ROI; red circle). HU values were obtained from the trabecular portion of the vertebral body, avoiding cortical margins and vascular channels

Quantitative attenuation measurements were performed independently by two radiologists, each with over 10 years of experience in abdominal imaging, who were blinded to the DXA results. Regions of interest (ROIs) were manually drawn on the central trabecular portion of the L1–L4 vertebral bodies on both sagittal and axial reconstructions, carefully excluding cortical bone, endplates, and vascular structures (Fig. 1). The ROI size was standardized, with an approximate surface area ranging between 80 and 150 mm^2^, adjusted according to vertebral body dimensions. For axial measurements, sagittal reformatted images were used as anatomical references to identify the true mid-vertebral body level before ROI placement. The mean HU value for each vertebra was recorded, and the average of L1–L4 was used as the representative vertebral attenuation for statistical analysis.

### DXA examination and classification

Bone mineral density was measured using a DXA scanner (Primus, Osteosys Co., Ltd., Guro-gu, Seoul, Republic of Korea), and *z*-scores for the lumbar spine (L1–L4) were calculated according to pediatric reference standards based on age and sex. In line with the 2019 ISCD Pediatric Official Positions (8), only *z*-scores ≤ − 2.0 were considered as low bone mineral density for chronological age. Because pediatric osteoporosis requires additional clinical criteria, the term “osteoporosis” was not used in this study.

### Statistical analysis

Statistical analyses were performed using SPSS version 22.0 (IBM Corp., Armonk, NY, USA). Normality of data distribution was assessed using the Shapiro–Wilk test. Continuous variables were expressed as mean ± standard deviation (SD) or median (interquartile range). Correlation between CT-derived mean HU values and DXA *z*-scores was analyzed using Pearson’s correlation coefficient. The correlation test results were interpreted as 0.0–0.20 (negligible), 0.21–0.40 (weak), 0.41–0.60 (moderate), 0.61–0.80 (strong), or 0.81–1.00 (very strong). The diagnostic performance of HU values in identifying low bone mineral density (*z* ≤ − 2.0) was evaluated with receiver operating characteristic (ROC) curve analysis. Area under the curve (AUC), optimal cut-off HU values, and corresponding sensitivity and specificity were calculated with 95% confidence intervals (CIs). A *p*-value < 0.05 was considered statistically significant.

## Results

### Patient characteristics

A total of 62 pediatric patients (mean age, 11.68 ± 3.20 years; range, 5–17 years) were included in the study. Of these, 27 patients (43.5%) were ≤ 10 years old and 35 (56.5%) were > 10 years. The cohort consisted of 38 males (61.3%) and 24 females (38.7%). The interval between CT and DXA examinations was limited to within 3 months according to the study inclusion criteria, with a mean interval of 0.97 ± 1.92 months. The mean lumbar DXA *z*-score was − 2.87 ± 1.48. Based on DXA classification, 24 patients (38.7%) had normal bone mineral density (*z* > − 2), whereas 38 patients (61.3%) were classified as having low bone mineral density (*z* ≤ − 2). When stratified by bone status, no significant differences were observed between the normal and low BMD groups with respect to mean age (*p* = 0.883), sex distribution (*p* = 0.498), interval between DXA and CT examinations (*p* = 0.812), or time since LT at the time of CT (*p* = 0.671). However, autoimmune liver diseases were significantly more frequent in the low BMD group compared with the normal BMD group (26.3% vs 8.3%, *p* = 0.028). Detailed demographic and clinical characteristics stratified by bone status are summarized in Table 1.

**Table 1:** Demographic and clinical characteristics

Variable	Normal BMD (*Z* > − 2.0) (*n* = 24)	Low BMD (*Z* ≤ − 2.0) (*n* = 38)	*p* value
**Age (years)**	11.2 ± 3.3	11.7 ± 3.1	0.883
**Male sex, *n* (%)**	14 (58.3%)	24 (63.2%)	0.498
**DXA *z*-score**	− 1.42 ± 0.46	− 3.79 ± 1.02	< 0.001
**Interval between DXA and CT (months)**	0.9 ± 1.8 (0–3)	1.0 ± 2.0 (0–3)	0.812
**Time since LT at CT (months)**	9.1 (7.0–12.0)	9.6 (6.5–13.0)	0.671
**Etiology, *n* (%)**			
• Cholestatic/biliary diseases	11 (45.8%)	17 (44.7%)	0.931
• Metabolic diseases	8 (33.3%)	12 (31.6%)	0.882
• Autoimmune liver diseases	2 (8.3%)	10 (26.3%)	0.028
• Cryptogenic	2 (8.3%)	5 (13.2%)	0.331
• Malignancy	1 (4.3%)	4 (10.5%)	0.301

*BMD* bone mineral density, *DXA* dual-energy X-ray absorptiometry, *CT* computed tomography, *LT* liver transplantation, *IQR* interquartile range

### CT attenuation measurements

**Fig. 2:**
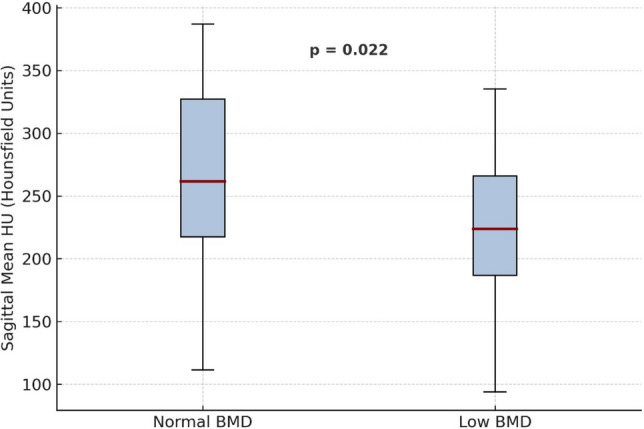
Comparison of mean sagittal CT–derived HU values between pediatric liver transplant recipients with normal and low BMD groups

Mean sagittal CT attenuation values were significantly lower in patients with low bone mineral density compared with those with normal bone mineral density at all lumbar levels (L1–L4, *p* < 0.05). The mean sagittal attenuation averaged across L1–L4 was 224.0 ± 46.5 HU in the low BMD group and 255.9 ± 59.5 HU in the normal BMD group (Fig. 2). In the axial plane, attenuation values were also lower in the low BMD group, but these differences did not reach statistical significance (*p* > 0.05). Detailed radiologic measurements are presented in Table 2.

**Table 2:** Sagittal and axial CT-derived HU measurements obtained from vertebral levels L1 to L4 in pediatric liver transplant recipients

Variable	Normal BMD (*N* = 24)	Low BMD (*N* = 38)	*p* value
Sagittal L1	254.8 ± 58.7	223.6 ± 49.0	**0.028**
Sagittal L2	256.1 ± 58.5	222.1 ± 47.1	**0.014**
Sagittal L3	254.5 ± 58.7	220.5 ± 44.8	**0.012**
Sagittal L4	258.3 ± 64.0	229.9 ± 49.1	**0.044**
Sagittal mean (L1–L4)	255.9 ± 59.5	224.0 ± 46.5	**0.022**
Axial L1	251.2 ± 63.3	230.3 ± 52.8	0.164
Axial L2	247.6 ± 67.0	220.6 ± 49.4	0.073
Axial L3	237.5 ± 61.9	219.1 ± 48.4	0.195
Axial L4	237.2 ± 63.9	224.6 ± 51.3	0.397
Axial mean (L1–L4)	243.4 ± 63.3	223.6 ± 49.3	0.175

Bold values indicate statistical significance (*p *0.05)

When analyzed by sex, males showed higher sagittal and axial attenuation values than females (all *p* < 0.05), although mean DXA *z*-scores and mean age did not differ significantly between sexes (*p* = 0.897 and *p* = 0.343, respectively).

### Correlation analyses and diagnostic performance

**Fig. 3:**
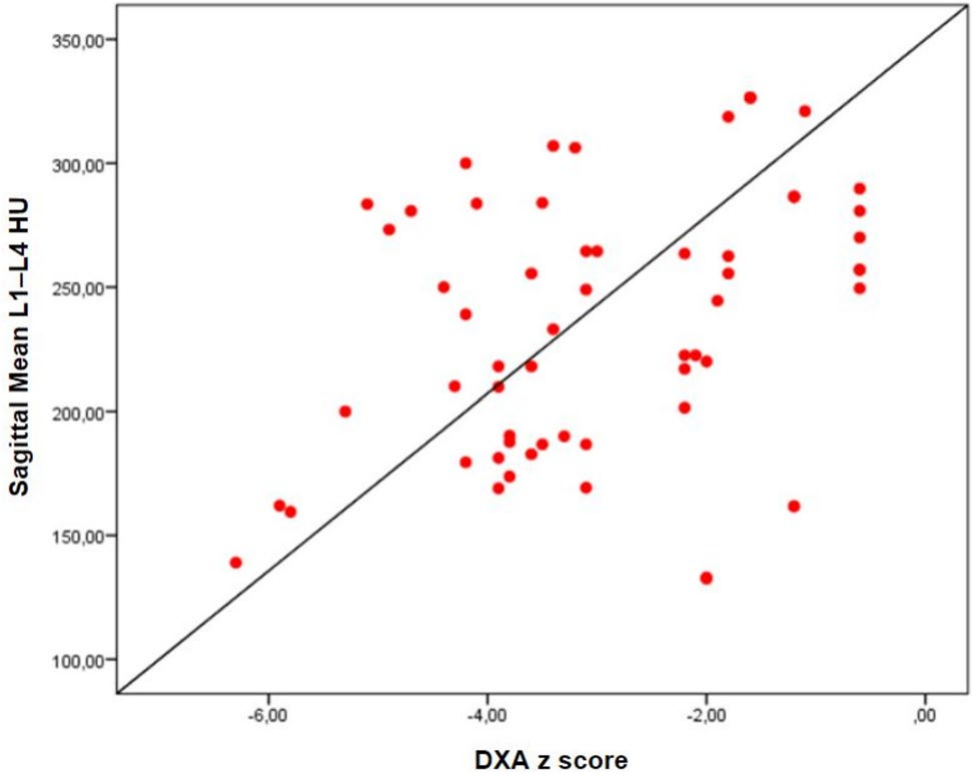
The correlation between mean sagittal CT–derived HU values measured across vertebral levels L1–L4 and DXA-derived *z*-scores in pediatric liver transplant recipients

Lumbar DXA *z*-scores correlated positively with CT attenuation values (Fig. 3). The sagittal mean HU showed a moderate positive correlation with BMD (*r* = 0.429, *p* = 0.003), whereas the axial mean HU demonstrated a weak correlation (*r* = 0.266, *p* = 0.037). Age demonstrated a significant negative correlation with mean CT-derived vertebral attenuation values. Specifically, age was moderately inversely correlated with mean sagittal HU values (*r* = − 0.385, *p* = 0.002) and mean axial HU values (*r* = − 0.406, *p* = 0.001). Mean HU measurements based on the sagittal and axial planes showed a very strong positive correlation (*r* = 0.977, *p* < 0.001). Additionally, HU measurements revealed robust inter-level correlations among lumbar vertebrae L1–L4 in both the sagittal (*r* = 0.941–0.971, all *p* < 0.001) and the axial (*r* = 0.934–0.968, all *p* < 0.001) planes.

**Fig. 4:**
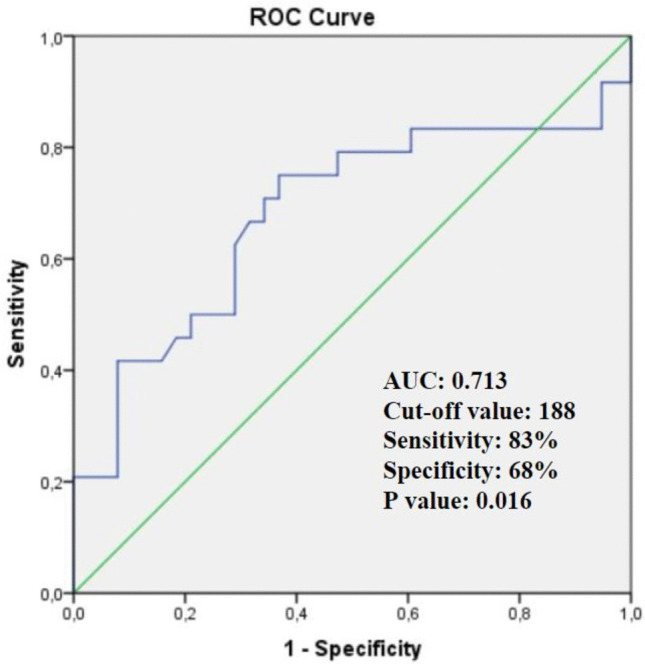
ROC curve illustrating the diagnostic performance of sagittal mean lumbar CT-derived HU values in identifying low BMD

Although statistically significant, the diagnostic performance of sagittal mean HU values was only moderate (AUC = 0.713, 95% CI 0.533–0.831, *p* = 0.016). Using an optimal cut-off value of approximately 188 HU, the sensitivity, specificity, positive predictive value (PPV), and negative predictive value (NPV) were 83.3%, 68.4%, 66.7%, and 84.2%, respectively, for distinguishing patients with low bone mineral density. In contrast, the axial mean HU demonstrated lower and statistically non-significant diagnostic performance (AUC = 0.627, 95% CI 0.474–0.780, *p* = 0.094) (Fig. 4).

## Discussion

As long-term survival rates after LT have improved, disorders of bone metabolism have gained increasing clinical relevance in pediatric recipients. Bone disease in children with chronic liver disease is associated with malnutrition, vitamin D deficiency, and cholestasis in the pretransplant period, while immunosuppressive medications and pubertal delay have been identified as additional risk factors in the posttransplant period [6, 13, 14]. Reported rates of low BMD in liver transplant recipients vary between 30 and 60%; Kotb et al. reported a low lumbar *z*-score in 48% of patients, and Valta et al. observed similar findings in 58% [7, 15]. In our cohort, 61% of patients demonstrated a lumbar *z*-score ≤ − 2, which is consistent with these previous reports. These results emphasize that systematic bone health surveillance should be considered an essential component of long-term follow-up in LT. In this context, the higher prevalence of autoimmune liver diseases in the low BMD group is clinically meaningful. Children with autoimmune hepatitis or immune-mediated cholangiopathies are often exposed to prolonged and repeated courses of corticosteroids, which are well known to adversely affect bone mineral accrual during growth. This finding supports the importance of closer skeletal surveillance in pediatric LT recipients with autoimmune etiologies.

Studies in the adult population have shown that HU values are lower in patients with osteopenia and osteoporosis than in individuals with normal BMD [16–20]. Our knowledge on this subject in the pediatric population is limited. In the literature, only Nagata et al. reported similar results in the adult population [21]. Our study found results consistent with those in the literature, with lower HU values in patients with low BMD. These findings support the idea that CT HU measurements can reflect bone mineral loss in children, as they do in adults, and could be a potential biomarker for non-invasive monitoring.

In the adult population, a significant correlation between CT HU and DXA was demonstrated. The correlation coefficients reported in the literature are generally moderate to strong (*r* = 0.44–0.75) [16–19, 22, 23]. In these studies, HU values measured at the lumbar spine and femoral neck exhibited a linear relationship with DXA *T*-scores and BMD values. However, different performance values (AUC ≈ 0.68–0.93) were reported for the diagnosis of osteoporosis. To our knowledge, our study is the first to examine the relationship between CT and DXA in pediatric liver transplant patients. Our findings indicate a moderate positive correlation (*r* = 0.42) between the mean HU value in the sagittal plane and the DXA *z*-score; however, the relationship was weaker in axial measurements (*r* = 0.27). ROC analysis revealed that mean HU in the sagittal plane had limited diagnostic value (AUC = 0.713) in predicting low BMD. These results are partially consistent with previous adult studies, suggesting that CT-derived HU values can serve as an auxiliary indicator of bone status. However, the moderate correlation and AUC observed in our pediatric transplant cohort indicate that CT-based assessment alone remains insufficient for reliable diagnosis and should be interpreted as a complementary rather than a standalone tool for bone health monitoring after liver transplantation.

One potential explanation for the low diagnostic sensitivity of CT HU measurements in our pediatric cohort is the dynamic and heterogeneous nature of the developing skeleton. In children, the proportion of trabecular bone is higher than in adults; however, this immature trabecular structure is not yet fully mineralized and undergoes substantial age-related changes [24]. The presence of open growth plates can further contribute to discrepancies between DXA-derived BMD and CT HU values, particularly before skeletal maturity is achieved. Moreover, pediatric bones typically exhibit thinner cortical thickness and a lower proportion of compact bone, which may diminish the strength of the association between HU measurements and true BMD [25].

Although a significant negative correlation was observed between age and CT-derived HU values, this finding should be interpreted with caution in pediatric liver transplant recipients. The bone health of this population is influenced by multiple interacting factors, including growth velocity, pubertal development, nutritional status, underlying liver disease, and long-term immunosuppressive therapy [3, 4]. Consequently, chronological age alone may not accurately reflect skeletal maturation or bone mineral accrual in this clinically complex group. In this study, CT-derived HU values were interpreted relative to DXA *z*-scores, which are inherently age-adjusted, consistent with our opportunistic imaging-based objective rather than the establishment of age-specific attenuation thresholds. In addition to physiological factors, the complex clinical context of LT—including surgical stress, immunosuppressive drugs, and nutritional alterations—may introduce secondary changes in bone architecture that are not fully captured by standard imaging metrics. In the present study, sagittal and axial HU measurements demonstrated a very strong positive correlation, indicating that both planes consistently reflect vertebral attenuation. However, despite this strong correlation, sagittal measurements showed lower variability and greater discriminatory ability between normal and low BMD groups, whereas axial measurements did not reach statistical significance. This difference in performance is therefore interpreted primarily as a reflection of measurement robustness rather than a fundamental discrepancy between imaging planes. Axial measurements may be more susceptible to partial volume effects, slice positioning, and anatomical variability, as they typically sample a more limited trabecular region, whereas sagittal reconstructions allow broader and more stable assessment of the vertebral body. These technical considerations have been noted in prior imaging studies [26, 27]. Furthermore, the strong inter-level correlations across L1–L4 suggest that a single-level HU measurement may provide a reasonable approximation of overall lumbar attenuation; however, averaging across multiple levels likely improves robustness by reducing measurement variability.

In addition, all HU measurements in this study were obtained from contrast-enhanced CT examinations, which may lead to higher vertebral attenuation values compared with non-contrast imaging. Previous studies have shown that intravenous contrast administration can influence trabecular bone attenuation, depending on contrast phase and vascular distribution [20, 27]. Although vessel-based normalization techniques using reference structures such as the aorta or portal vein have been proposed to reduce contrast-related variability, the primary objective of our analysis was to evaluate an opportunistic imaging approach that could be readily applied in routine clinical practice to support rapid clinical decision-making without additional post-processing. Incorporating ratio-based measurements requiring multiple reference ROIs would have increased analysis time and complexity, potentially limiting the practicality of this approach in daily clinical workflows.

This study has several limitations. Firstly, the retrospective and single-center design of the study limits the generalisability of the findings. Secondly, despite the use of standardized imaging protocols, CT-derived HU measurements may still be affected by technical factors such as scanner calibration, the timing of contrast administration, and patient body habitus. All HU measurements were obtained from contrast-enhanced CT examinations, which may have influenced absolute attenuation values. Additionally, the interval of up to 3 months between DXA and CT examinations may have introduced temporal variability. However, a shorter interval would have substantially reduced the sample size in this clinically specific pediatric transplant cohort. Finally, the analysis was limited to imaging data, so detailed clinical factors influencing bone health could not be assessed. Accordingly, the study was designed to evaluate the relationship between DXA *z*-scores and CT-derived HU values, rather than establishing causal clinical associations or age-specific reference thresholds.

## Conclusion

CT-derived HU measurements obtained from routine abdominal imaging appear to offer meaningful adjunctive insights into the evaluation of bone health among pediatric liver transplant recipients. Despite their potential to opportunistically reflect bone mineral status without additional radiation exposure or cost, the observed moderate correlation and limited diagnostic concordance with DXA indicate that CT cannot substitute for DXA as the reference standard. Instead, CT-based bone assessment should be considered a complementary approach, enhancing clinical decision-making by providing supportive quantitative information in scenarios where DXA is unavailable or impractical.

## Data Availability

The data that support the findings of this study are stored at Inonu University, Liver Transplant Institute, Malatya, Türkiye, and it can be requested from the corresponding author upon reasonable request.

## Abbreviations

BMD: Bone mineral density
CT: Computerized tomography
DXA: Dual-energy X-ray absorptiometry
HU: Hounsfield unit
ISCD: International Society for Clinical Densitometry
LT: Liver transplantation

## References

[1] Dag N, Karatoprak S, Ozturk M, Karatoprak NB, Sigirci A, Yilmaz S (2021) Investigation of the prognostic value of psoas muscle area measurement in pediatric patients before liver transplantation: a single-center retrospective study. Clin Transplant 35(10):e14416. 10.1111/ctr.14416 34231257 10.1111/ctr.14416

[2] Lee S, Lee S-K (2019) Pediatric liver transplantation in Korea: long-term outcomes and allocations. J Korean Soc Transplant 33(1):1–5. 10.4285/jkstn.2019.33.1.1

[3] Högler W, Baumann U, Kelly D (2012) Endocrine and bone metabolic complications in chronic liver disease and after liver transplantation in children. J Pediatr Gastroenterol Nutr 54(3):313–321. 10.1097/MPG.0b013e31823e9412 22064631 10.1097/MPG.0b013e31823e9412

[4] Dag N, Ozturk M, Sigirci A, Yilmaz S (2022) Scoliosis after liver transplantation in pediatric patients. Eur J Ther. 10.5152/EurJTher.2022.21081

[5] Danford CJ, Trivedi HD, Bonder A (2020) Bone health in patients with liver diseases. J Clin Densitom 23(2):212–222. 10.1016/j.jocd.2019.01.004 30744928 10.1016/j.jocd.2019.01.004

[6] Rodríguez-Aguilar EF, Pérez-Escobar J, Herrera DS, García-Alanis M, Toapanta-Yanchapaxi L, Gonzalez-Flores E et al editors (2021) Bone disease and liver transplantation: a review. Transplant Proc 10.1016/j.transproceed.2021.07.049 10.1016/j.transproceed.2021.07.049 34420781

[7] Valta H, Jalanko H, Holmberg C, Helenius I, Mäkitie O (2008) Impaired bone health in adolescents after liver transplantation. Am J Transplant 8(1):150–157. 10.1111/j.1600-6143.2007.02015.x 17973968 10.1111/j.1600-6143.2007.02015.x

[8] Shuhart CR, Yeap SS, Anderson PA et al (2019) Executive summary of the 2019 ISCD position development conference on monitoring treatment, DXA cross-calibration and least significant change, spinal cord injury, peri-prosthetic and orthopedic bone health, transgender medicine, and pediatrics. J Clin Densitom 22(4):453–71. 10.1016/j.jocd.2019.07.001 31400968 10.1016/j.jocd.2019.07.001

[9] Weber DR, Boyce A, Gordon C et al (2019) The utility of DXA assessment at the forearm, proximal femur, and lateral distal femur, and vertebral fracture assessment in the pediatric population: 2019 ISCD official position. J Clin Densitom 22(4):567–89. 10.1016/j.jocd.2019.07.002 31421951 10.1016/j.jocd.2019.07.002 PMC7010480

[10] Ofori EK, Amponsah SK (2025) Dual X-ray absorptiometry: a backbone. Diagn Adv Precis Med Drug Dev 67. 10.1201/9781003486909-6

[11] Öztürk M, Dağ N, Sığırcı A, Yılmaz S (2021) Evaluation of early and late complications of pediatric liver transplantation with multi-slice computed tomography: a high-volume transplant single-center study. Turk J Gastroenterol 32(7):586. 10.5152/tjg.2021.20564 34464322 10.5152/tjg.2021.20564 PMC8975438

[12] Ahmad A, Crawford IIICH, Glassman SD, Dimar IIJR, Gum JL, Carreon LY (2023) Correlation between bone density measurements on CT or MRI versus DEXA scan: a systematic review. N Am Spine Soc J 14:100204. 10.1016/j.xnsj.2023.100204 37090222 10.1016/j.xnsj.2023.100204 PMC10119682

[13] Astolfi D, Rock N, Ceroni D, Wildhaber BE (2024) Predictors for pathological bone fractures in children undergoing liver transplantation: a retrospective cohort study. Pediatr Transplant 28(3):e14755. 10.1111/petr.14755 38623895 10.1111/petr.14755

[14] Ballan D, Luisetto G, Cillo U, Guariso G, Zancan L (2004) Long-term outcome of bone mineral density in children who underwent a successful liver transplantation. Transplantation 78(6):899–903. 10.1097/01.tp.0000136987.38729.c0 15385811 10.1097/01.tp.0000136987.38729.c0

[15] Kotb MA, Fawaz LA, Zeitoun RA et al (2022) Bone demineralization in a cohort of Egyptian pediatric liver transplant recipients: Single center pilot study. Medicine 101(45):e31156. 10.1097/MD.0000000000031156 36397404 10.1097/MD.0000000000031156 PMC10662835

[16] Alpaslan M, Ozkacmaz S, Bora A (2020) The comparison of computed tomography densitometry and DEXA for diagnosis of osteoporosis. Ann Clin Anal Med 11(3). 10.4328/acam.20041

[17] Courtois EC, Ohnmeiss DD, Guyer RD (2023) Assessing lumbar vertebral bone quality: a methodological evaluation of CT and MRI as alternatives to traditional DEXA. Eur Spine J 32(9):3176–3182. 10.1007/s00586-023-07855-6 37439864 10.1007/s00586-023-07855-6

[18] Choi MK, Kim SM, Lim JK (2016) Diagnostic efficacy of Hounsfield units in spine CT for the assessment of real bone mineral density of degenerative spine: correlation study between T-scores determined by DEXA scan and Hounsfield units from CT. Acta Neurochir 158(7):1421–1427. 10.1007/s00701-016-2821-5 27177734 10.1007/s00701-016-2821-5

[19] Alawi M, Begum A, Harraz M et al (2021) Dual-energy X-ray absorptiometry (DEXA) scan versus computed tomography for bone density assessment. Cureus. 10.7759/cureus.13261 33717764 10.7759/cureus.13261 PMC7954087

[20] Cohen A, Foldes AJ, Hiller N, Simanovsky N, Szalat A (2021) Opportunistic screening for osteoporosis and osteopenia by routine computed tomography scan: a heterogeneous, multiethnic, Middle-Eastern population validation study. Eur J Radiol 136:109568. 10.1016/j.ejrad.2021.109568 33545629 10.1016/j.ejrad.2021.109568

[21] Nagata K, Glassman SD, Dimar JR et al (2024) Comparison of bone mineral density in children and adolescents on ct versus dexa scan. Spine 49(19):E322–E6. 10.1097/BRS.0000000000004877 37970684 10.1097/BRS.0000000000004877

[22] Francisco I, Nunes C, Pereira F et al (2023) Bone mineral density through DEXA and CBCT: a systematic review with meta-analysis. Appl Sci 13(10):5962. 10.3390/app13105962

[23] Chia KK, Haron J, Malek NFSN (2021) Accuracy of computed tomography attenuation value of lumbar vertebra to assess bone mineral density. Malays J Med Sci 28(1):41. 10.21315/mjms2021.28.1.6 33679219 10.21315/mjms2021.28.1.6 PMC7909356

[24] Farr JN, Amin S, LeBrasseur NK et al (2014) Body composition during childhood and adolescence: relations to bone strength and microstructure. J Clin Endocrinol Metab 99(12):4641–8. 10.1210/jc.2014-1113 25243571 10.1210/jc.2014-1113 PMC4255129

[25] Sioen I, Lust E, De Henauw S, Moreno L, Jiménez-Pavón D (2016) Associations between body composition and bone health in children and adolescents: a systematic review. Calcif Tissue Int 99(6):557–77. 10.1007/s00223-016-0183-x 27484027 10.1007/s00223-016-0183-x

[26] Di Iorgi N, Maruca K, Patti G, Mora S (2018) Update on bone density measurements and their interpretation in children and adolescents. Best Pract Res Clin Endocrinol Metab 32(4):477–498. 10.1016/j.beem.2018.06.002 30086870 10.1016/j.beem.2018.06.002

[27] Pickhardt PJ, Pooler BD, Lauder T, del Rio AM, Bruce RJ, Binkley N (2013) Opportunistic screening for osteoporosis using abdominal computed tomography scans obtained for other indications. Ann Intern Med 158(8):588–95. 10.7326/0003-4819-158-8-201304160-00003 23588747 10.7326/0003-4819-158-8-201304160-00003 PMC3736840

